# Supplementary material for: Association of HSV-1 and Reduced Oral Bacteriota Diversity with Chemotherapy-Induced Oral Mucositis in Patients Undergoing Autologous Hematopoietic Stem Cell Transplantation
Source: J Clin Med. 2020 Apr 11;9(4):1090. doi: 10.3390/jcm9041090 (PMC7230275; doi:10.3390/jcm9041090)
Supplement: Supplementary file 1 [file jcm-09-01090-s001.pdf]

Supplementary Materials:

Table S1. Information on autologous stem cell transplantation in analyzed patients.

Type of disease	Autologous HSCT regimen	Day of stem cell infusion
Multiple myeloma (*n* = 28)	High dose melphalan (n = 16):<br>*Melphalan 100 mg/m^2/day for 2 consecutive days (day 0~1)*	Day 4
	Thiotepa, busulfan, and cyclophosphamide (TBC; n = 6):<br>*Thiotepa 200 mg/m^2/day for 3 consecutive days (days 0~2)*<br>*Busulfan 3.2 mg/kg/day for 3 consecutive days (days 3~4)*<br>*Cyclophosphamide 50 mg/kg for 2 consecutive days (days 5~6)*	Day 8
	Busulfan, cyclophosphamide, and etoposide (BuEtoCy; n = 3):<br>*Busulfan 3.2 mg/kg/day for 3 consecutive days (days 0~2)*<br>*Etoposide 400 mg/m^2 for 2 consecutive days (days 2~3)*<br>*Cyclophosphamide 50 mg/kg for 2 consecutive days (days 4~5)*	Day 7
	Melphalan and busulfan (BuMel; n = 3):<br>*Busulfan 3.2 mg/kg/day for 3 consecutive days (days 0~2)*<br>*Melphalan 70 mg/m^2/day for 2 consecutive days (days 3~4)*	Day 6
Malignant lymphoma (*n* = 15)<br>1) Diffuse large B-cell lymphoma (*n* = 7)<br>2) Peripheral T-cell lymphoma (*n* = 7)<br>3) Hodgkin lymphoma (*n* = 1)	BuEtoCy (n = 8)	Day 7
	TBC (n = 6)	Day 8
	Busulfan, etoposide, cytarabine, and melphalan (BuEAM; n = 1):<br>*Busulfan 3.2 mg/kg/day for 3 consecutive days (days 0~2)*<br>*Etoposide 200 mg/m^2/day for 2 consecutive days (days 2~3)*<br>*Cytarabine 1 gram/m^2/12 hour for 2 consecutive days (days 2~3)*<br>*Melphalan 140 mg/m^2/day on day 4*	Day 6
Germ cell tumor (*n* = 1)	Carboplatin, thiotepa, and etoposide:<br>*Carboplanin 500 mg/m^2/day for 3 consecutive days (days 0~2)*<br>*Thiotepa 300 mg/m^2/day for 3 consecutive days (days 3~5)*<br>*Etoposide 250 mg/m^2/day for 3 consecutive days (days 3~5)*	Day 8
Rhabdomyosarcoma (*n* =1)	Etoposide and carboplatin:<br>*Carboplatin 700 mg/m^2/day for 3 consecutive days (days 0~2)*<br>*Etoposide 750 mg/m^2/day for 3 consecutive days (days 0~2)*	Day 5
Retractable thrombocytopenia (*n* = 1)	Cyclophosphamide, fludarabine, anti-thymocyte globulin (ATG), and methylprednisolone:<br>*Fludarabine 30 mg/m^2/day for 5 consecutive days (days 0~4)*<br>*ATG 2.5 mg/kg/day for 3 consecutive days (days 4~6)*<br>*Methylprednisolone 1 mg/kg/day for 3 consecutive days (days 4~6)*<br>*Cyclophosphamide 50 mg/kg for 2 consecutive days (days 4~5)*	Day 7

Table S2. Relative abundances of species/phylotypes that are differentially distributed among controls, the baseline and post-chemotherapy communities of patients.

**Name**	Controls n = 15	Patients n = 46		p-values[b]	
		Baseline	Post-CTx	Control vs. Baseline[c]	Baseline vs. Post-CTx[d]
Scardovia wiggsiae	**0 (0.14)**	**0.015 (10.81)**	0.001 (9.09)	**0.036**	0.407
Actinomycetaceae_uc_s	**0 (0.01)**	**0.010 (0.38)**	**0 (0.48)**	**0.036**	**0.013**
Micrococcaceae_uc_s	**0 (0.01)**	**0.010 (0.29)**	0.002 (0.40)	**<0.001**	0.407
Mycoplasma salivarium	**0 (0.08)**	**0.007 (4.13)**	**0.167 (59.10)**	**0.036**	**0.002**
Actinomyces israelii	**0 (0.01)**	**0.005 (0.08)**	0 (0.25)	**0.036**	0.407
Staphylococcus epidermidis	**0 (0.02)**	**0.005 (1.65)**	0.010 (78.48)	**<0.001**	0.407
Flavobacteriaceae_uc_s	**0 (0)**	**0 (0.17)**	**0 (0.18)**	**0.036**	**0.048**
Prevotellaceae_uc_s	**0 (0.01)**	**0.003 (0.14)**	0.001 (0.14)	**0.036**	0.407
Aggregatibacter sp. HOT 458	**0.219 (0.55)**	**0 (2.62)**	0 (0.27)	**<0.001**	0.093
Neisseria oralis	**0.180 (7.16)**	**0 (41.83)**	0 (85.45)	**0.036**	0.342
Bergeyella sp. HOT 322	**0.163 (0.98)**	**0.020 (0.19)**	0 (0.31)	**<0.001**	0.302
Haemophilus sputorum	**0.134 (8.25)**	**0 (18.35)**	0 (0.14)	**0.036**	0.407
Alloprevotella sp. HOT 914	**0.103 (0.57)**	**0 (0.61)**	0 (1.44)	**0.036**	0.344
Leptotrichia hofstadii	**0.034 (0.22)**	**0 (0.1)**	0 (0.64)	**0.036**	0.228
Aggregatibacter segnis	**0.025 (0.45)**	**0 (0.50)**	0 (0.11)	**0.036**	0.407
Haemophilus parainfluenzae	6.280 (33.30)	**0.330 (13.01)**	**0.003 (8.88)**	0.887	**0.023**
Streptococcus salivarius	2.063 (8.12)	**0.766 (18.03)**	**0.006 (13.79)**	0.485	**0.007**
Streptococcus sanguinis	1.573 (20.77)	**1.244 (19.40)**	**0.006 (6.81)**	0.887	**<0.001**
Rothia aeria	0.711 (7.27)	**0.218 (8.39)**	**0.004 (8.34)**	0.309	**0.004**
Streptococcus sinensis	0.658 (7.46)	**0.627 (7.19)**	**0.001 (47.10)**	0.961	**0.020**
Fusobacterium periodonticum	0.556 (1.54)	**0.051 (5.86)**	**0 (1.46)**	1	**0.003**
Actinomyces_uc	0.484 (13.27)	**0.198 (6.97)**	**0.018 (5.65)**	0.978	**0.016**
Gemella haemolysans	0.476 (2.29)	**0.122 (15.60)**	**0.001 (12.56)**	0.887	**0.023**
Corynebacterium durum	0.335 (2.03)	**0.034 (8.39)**	**0 (14.88)**	0.485	**0.022**
Streptococcus parasanguinis	0.318 (3.95)	**0.409 (7.90)**	**0.006 (1.26)**	0.887	**0.002**
Prevotella melaninogenica	0.308 (2.92)	**0.117 (45.50)**	**0.003 (2.81)**	0.797	**0.019**
Leptotrichia hongkongensis	0.212 (1.92)	**0.154 (21.03)**	**0 (1.06)**	1	**0.001**
Actinomyces odontolyticus	0.173 (2.19)	**0.037 (1.88)**	**0 (0.54)**	0.211	**0.049**
Prevotella oris	0.160 (2.56)	**0.199 (2.61)**	**0 (2.91)**	1	**0.019**
Actinomyces naeslundii	0.160 (1.07)	**0.247 (52.27)**	**0.008 (2.97)**	0.887	**0.020**
Actinomyces viscosus	0.153 (1.46)	**0.500 (14.07)**	**0.015 (8.72)**	0.142	**0.004**
Capnocytophaga sputigena	0.129 (2.20)	**0.189 (8.06)**	**0.001 (14.19)**	0.961	**0.012**
Haemophilus_uc	0.123 (1.58)	**0 (1.22)**	**0 (0.05)**	0.052	**0.010**
Capnocytophaga leadbetteri	0.088 (0.41)	**0.189 (17.45)**	**0 (2.23)**	0.887	**0.008**

Actinomyces oris	0.083 (4.29)	**0.063 (10.13)**	**0 (0.70)**	1	**0.001**
Capnocytophaga granulosa	0.060 (1.07)	**0.353 (6.32)**	**0.002 (13.72)**	0.166	**0.021**
Oribacterium sinus	0.054 (0.64)	**0.113 (1.49)**	**0 (0.33)**	0.747	**<0.001**
Corynebacterium matruchotii	0.054 (1.38)	**0.097 (6.32)**	**0 (4.32)**	0.887	**0.004**
Stomatobaculum longum	0.053 (0.34)	**0.081 (1.07)**	**0 (0.21)**	0.556	**<0.001**
Cardiobacterium hominis	0.050 (0.70)	**0.026 (1.45)**	**0 (0.95)**	0.887	**0.013**
Leptotrichia sp. HOT 463	0.045 (1.56)	**0.131 (8.35)**	**0 (0.32)**	0.887	**0.002**
Veillonella atypica	0.041 (0.97)	**0.096 (3.56)**	**0 (23.43)**	0.887	**0.007**
Lachnoanaerobaculum umeaense	0.037 (0.32)	**0.020 (0.60)**	**0 (0.18)**	0.887	**0.002**
Neisseria flava	0.037 (7.85)	**0.294 (92.16)**	**0.007 (47.02)**	0.490	**0.049**
Streptococcus intermedius	0.036 (0.46)	**0.065 (1.97)**	**0.002 (0.43)**	0.618	**0.035**
Neisseria elongata	0.033 (1.60)	**0.017 (5.47)**	**0 (21.48)**	1	**0.030**
Prevotella pallens	0.031 (0.44)	**0.021 (1.43)**	**0 (0.33)**	1	**0.004**
Capnocytophaga gingivalis	0.030 (0.43)	**0.024 (1.54)**	**0 (1.70)**	1	**0.002**
Capnocytophaga ochracea	0.023 (0.23)	**0.008 (1.09)**	**0 (1.07)**	1	**0.004**
Streptococcus peroris	0.022 (1.71)	**0.073 (2.16)**	**0.002 (0.23)**	0.887	**0.002**
Capnocytophaga_uc	0.021 (2.02)	**0.089 (2.31)**	**0.004 (1.68)**	0.235	**0.004**
Leptotrichia sp. HOT 219	0.020 (0.17)	**0.001 (2.13)**	**0 (0.05)**	0.887	**0.017**
Gemella morbillorum	0.020 (0.15)	**0 (2.09)**	**0 (0.13)**	0.887	**0.043**
Catonella morbi	0.019 (0.20)	**0.024 (0.77)**	**0 (0.22)**	0.887	**0.013**
Prevotella_uc	0.018 (0.51)	**0.060 (0.52)**	**0.003 (0.31)**	0.566	**0.006**
Selenomonas noxia	0.017 (0.74)	**0.031 (2.59)**	**0 (0.59)**	1	**0.009**
Eubacterium sulci	0.014 (0.08)	**0 (0.15)**	**0 (0.07)**	0.887	**0.034**
Prevotella histicola	0.013 (0.49)	**0.039 (9.66)**	**0 (25.67)**	0.887	**0.008**
Prevotella oulorum	0.012 (0.29)	**0.020 (0.84)**	**0 (0.29)**	1	**0.025**
Peptidiphaga gingivicola	0.011 (0.43)	**0.005 (0.92)**	**0 (4.32)**	1	**0.038**
Capnocytophaga sp. HOT 864	0.011 (0.19)	**0.001 (1.89)**	**0 (0.52)**	0.979	**0.038**
Leptotrichia sp. HOT 215	0.011 (0.11)	**0 (0.74)**	**0 (0.09)**	1	**0.035**
Streptococcus gordonii	0.009 (5.21)	**0.126 (4.11)**	**0 (12.76)**	0.806	**0.006**
Solobacterium moorei	0.007 (0.15)	**0.024 (0.38)**	**0 (0.28)**	0.705	**0.019**
Alloprevotella sp. HOT 308	0.006 (0.24)	**0.041 (0.95)**	**0 (0.65)**	0.485	**0.043**
Actinomyces graevenitzii	0.006 (0.35)	**0.020 (1.10)**	**0 (0.83)**	0.975	**0.001**
Corynebacterium matruchotii	0.005 (1.25)	**0.006 (3.51)**	**0 (0.54)**	0.887	**0.022**
Ottowia sp. HOT 894	0.005 (0.60)	**0.002 (2.53)**	**0 (0.38)**	1	**0.016**
Lachnoanaerobaculum orale	0.004 (0.16)	**0.058 (3.45)**	**0 (0.11)**	0.410	**0.002**
Capnocytophaga sp. HOT 864	0.004 (0.24)	**0.008 (1.75)**	**0 (3.90)**	1	**0.040**
Fusobacterium_uc	0.003 (0.57)	**0.023 (0.44)**	**0.002 (0.29)**	0.751	**0.017**
Haemophilus pittmaniae	0.003 (13.91)	**0 (2.13)**	**0 (0.02)**	0.539	**0.050**
Selenomonas sputigena	0.002 (0.28)	**0.038 (1.10)**	**0 (0.18)**	0.534	**0.002**
Actinomyces bowdenii	0.002 (0.07)	**0.010 (0.34)**	**0 (0.06)**	0.743	**0.003**
Leptotrichia shahii	0 (0.17)	**0.019 (5.41)**	**0 (0.53)**	0.714	**0.038**
Selenomonas sp. HOT 136	0 (0.06)	**0.015 (3.08)**	**0 (0.72)**	0.173	**0.048**
Selenomonas_uc	0 (0.21)	**0.013 (0.46)**	**0 (0.07)**	0.214	**0.006**
Leptotrichia_uc	0 (0.01)	**0.009 (0.72)**	**0 (0.03)**	0.235	**0.002**
Selenomonas dianae	0 (0.08)	**0.005 (0.56)**	**0 (0.02)**	0.485	**0.001**
Stomatobaculum sp. HOT 097	0 (0.04)	**0.005 (0.36)**	**0 (0.03)**	0.986	**0.002**
Centipeda periodontii	0 (0.10)	**0.004 (0.23)**	**0 (0.42)**	0.618	**0.025**
Veillonellaceae sp. HOT 155	0 (0.12)	**0.003 (0.82)**	**0 (0.46)**	0.887	**0.015**
Anaeroglobus geminatus	0 (0.03)	**0.002 (0.77)**	**0 (0.36)**	0.485	**0.039**
Porphyromonas_uc	0 (0.01)	**0.001 (0.51)**	**0 (0.14)**	0.166	**0.040**
Leptotrichia sp. HOT 221	0 (0.21)	**0 (1.89)**	**0 (0.03)**	0.887	**0.008**

Leptotrichiaceae_uc_s	0 (0)	**0 (0.39)**	**0 (0.01)**	0.076	**0.013**
Corynebacteriaceae_uc_s	0 (0)	**0 (0.12)**	**0 (0)**	0.110	**0.017**
Streptococcus mutans	0 (0.10)	**0 (2.46)**	**0 (0.37)**	0.968	**0.017**
Gemella_uc	0 (0.01)	**0 (0.23)**	**0 (0.09)**	0.173	**0.019**
Selenomonas flueggei	0 (0.02)	**0 (0.26)**	**0 (0.01)**	0.462	**0.020**
Corynebacterium_uc	0 (0.05)	**0 (0.33)**	**0 (0.03)**	0.887	**0.020**
Lachnoanaerobaculum_uc	0 (0.01)	**0 (0.06)**	**0 (0)**	0.587	**0.020**
Bacteroidales_uc_s	0 (0.01)	**0 (0.01)**	**0 (0.01)**	0.789	**0.023**
Pasteurellaceae_uc_s	0 (0.03)	**0 (0.42)**	**0 (0.02)**	0.887	**0.024**
Prevotella veroralis	0 (0.28)	**0 (0.66)**	**0 (0.29)**	0.913	**0.028**
Dialister pneumosintes	0 (0.09)	**0 (1.01)**	**0 (0.41)**	0.887	**0.033**
Veillonellaceae sp. HOT 145	0 (0.02)	**0 (0.50)**	**0 (0.06)**	0.383	**0.040**
Prevotella sp. HOT 314	0 (0.43)	**0 (1.55)**	**0 (0.03)**	1	**0.045**
Leptotrichia trevisanii	0 (0.04)	**0 (0.72)**	**0 (0.02)**	0.887	**0.046**
Fretibacterium sp. HOT 360	0 (0.12)	**0 (1.00)**	**0 (0.15)**	1	**0.046**
Selenomonas sp. HOT 137	0 (0.01)	**0 (0.46)**	**0 (0.12)**	0.485	**0.048**
Cardiobacterium valvarum	0 (0.07)	**0 (0.27)**	**0 (0.18)**	0.887	**0.048**
Hallella seregens	0 (0.10)	**0 (0.52)**	**0 (0.34)**	0.887	**0.048**
Aggregatibacter_uc	0 (0.04)	**0 (0.34)**	**0 (0)**	0.887	**0.049**

[a] Relative abundance is expressed as median (range). [b] *P* values were adjusted using the Benjamini-Hochberg false discovery rate method. [c] By Mann-Whitney U test; [d] By Wilcoxon signed-rank test. Bold denotes statistical significance.

Table S3. The effect of the use of acyclovir on post-chemotherapy HSV-1 positivity and OM development.

		Post-CTx HSV-1		*p*	OM experience		*P*
		Negative	Positive		No	Yes	
The use of acyclovir	No	14 (73.7%)	20 (74.1%)	0.618	20 (76.9%)	14 (70.0%)	0.738
	Yes	5 (26.3%)	7 (25.9%)		6 (23.1%)	6 (30.0%)	

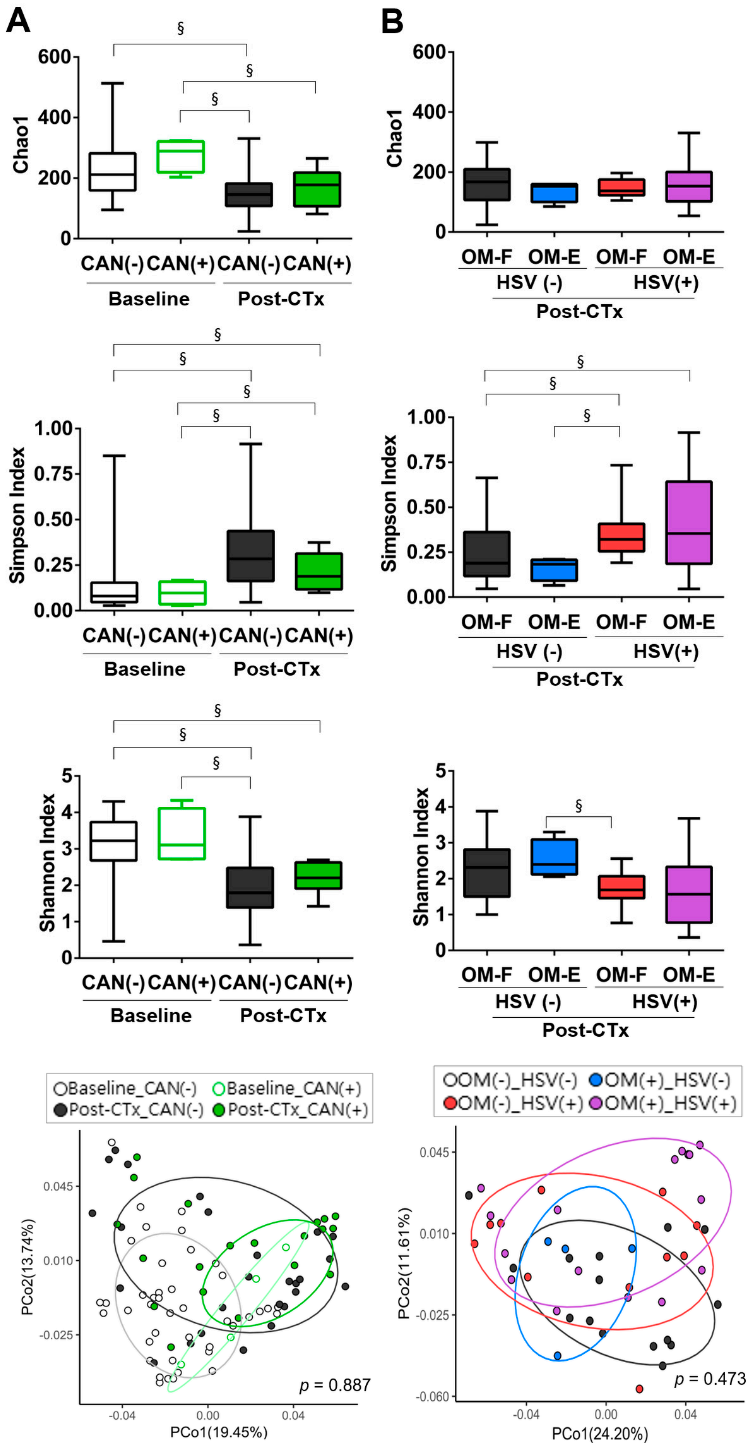

Figure S1. Structures of the mucosal bacterial communities collected from patients undergoing autologous HSCT at baseline and post-chemotherapy (post-CTx). (**A**) The baseline and post-chemotherapy communities without or with Candida spp. were compared. (**B**) The post-chemotherapy communities divided into four groups by the presence of HSV-1 and OM were compared.
